# Spinal myxopapillary ependymoma in an adult male presenting with recurrent acute low back pain: a case report

**DOI:** 10.1186/s12998-016-0094-y

**Published:** 2016-04-18

**Authors:** Dean Petersen, Reidar P. Lystad

**Affiliations:** Private practice, Mackay, QLD Australia; Department of Chiropractic, Macquarie University, Sydney, Australia

**Keywords:** Myxopapillary ependymoma, Spinal cord, Filum terminale, Neoplasm, Chiropractic

## Abstract

**Background:**

Spinal intramedullary ependymomas are very rare and occur more commonly in the cervical and upper thoracic regions. These neoplasms tend to manifest in young adulthood, and patients typically present with mild clinical symptoms without objective evidence of neurologic deficits. The mean duration of symptoms is 40 months until the lesion is diagnosed.

**Case Presentation:**

A 48-year-old male police officer was referred to a chiropractic clinic by a general practitioner for the evaluation of recurrent acute low back pain (LBP). Although the first episode of LBP was resolved, the clinical examination during the second episode revealed subtle changes that warranted referral to magnetic resonance imaging (MRI). The MRI revealed a spinal myxopapillary ependymoma.

**Conclusion:**

Because the primary symptoms of spinal intramedullary ependymomas can mimic ordinary LBP presentations, in particular lumbar intervertebral disc herniations, clinicians need to be sensitive to subtle changes in the clinical presentation of LBP patients. Prompt referral to advanced medical imaging such as MRI and early neurosurgical intervention is key to achieve best possible outcomes for patients with spinal intramedullary ependymomas.

## Background

Primary intraspinal neoplasms are rare, with an age-adjusted incidence rate of 0.5 in females and 0.3 in males per 100,000 population per annum [1]. Ependymoma is the most common intramedullary spinal neoplasm in adults, accounting for up to 60 % of all glial spinal cord tumors [2]. Spinal ependymomas occur most commonly in the cervical and upper thoracic regions, and only 6.5 % involve either the distal thoracic cord or the conus medullaris [2, 3]. Myxopapillary ependymoma, a benign special variant of ependymoma that is thought to arise from ependymocytes of the filum terminale, constitutes approximately 13 % of all spinal ependymomas in this region [4].

Spinal myxopapillary ependymomas tend to manifest in adulthood (mean age, 35 years) and is more commonly seen in male patients [2]. Patients typically present with mild clinical symptoms without objective evidence of neurologic deficits, which often results in a delay in diagnosis (mean duration of symptoms is 37 to 42 months) [2, 5]. At the time of diagnosis, patients typically have back or neck pain (67 %), sensory deficits (52 %), motor weakness (46 %), or bowel or bladder dysfunction (15 %) [2, 3]. Importantly, patients with a shorter duration of symptoms and less preoperative neurologic deficit tend to have better postoperative outcomes [2, 3].

Thus it becomes vital to promptly recognise important clues in the clinical presentation and evaluation that will lead to early diagnosis. Here we describe a rare case of an adult male with spinal myxopapillary ependymoma presenting as recurrent acute low back pain with subtle neurologic symptoms. The clinical presentation, chiropractic examination and management, imaging manifestations, and subsequent neurosurgical intervention and outcome are discussed below.

## Case presentation

### Clinical history

A 48-year-old male police officer was referred to a chiropractic clinic by a general practitioner for the evaluation of acute low back pain (LBP) of two weeks duration. The patient reported that the LBP had come on after spending a 12-h night shift driving a patrol car. The LBP was described as diffuse and the patient reported sharp pain radiating down the left lateral thigh to the knee. The average pain intensity was rated as 6/10 on a visual analogue scale (VAS), but had progressed to 8/10 VAS at the time of presentation. The symptoms were aggravated by sitting, sleeping on the left side, and inactivity; while standing provided the most relief. The pain was worse in the morning, and improved only marginally during the day. Self-care with heat packs and over-the-counter non-steroidal anti-inflammatory drugs (6 × 200 mg ibuprofen tablets per day) provided only a 2-point reduction on the VAS. The patient denied the presence of any paraesthesia (e.g. pins or needles), saddle anaesthesia, loss or changes in bladder or bowel control, fever, or nocturnal pain. The patient reported an approximately 20 kg reduction in body weight over the past 10 months, which he attributed to dietary changes and uptake of a substantial exercise regimen.

The patient also complained of left-sided headaches in the suboccipital and temporal regions of three weeks duration. The headaches were accompanied by occasional dizziness (clarified to be a feeling of unease) that were aggravated by sustained awkward neck postures. He had a family history of hypertension, type 2 diabetes, and migraines. His grandmother had passed away from bowel cancer. The patient was currently being treated for haemochromotosis, for which he had been managed successfully for 8 years. The rest of the systems review was unremarkable.

### Physical examination

The physical examination revealed a marked antalgic posture to the right with limited active lumbar range of motion (ROM) in left lateral flexion and flexion (finger-to-floor reach was noted to be 2 cm above the knees). Coughing, sneezing, and Valsalva manoeuvre reproduced the LBP, but not the leg pain. Straight leg raise was positive for leg and back pain at 40° on the left and negative at 70° on the right. Bragard’s test was positive on the left, while slump test and well leg raise were negative. Deep tendon reflexes were normal (2+) in both upper and lower limbs, except for the patella reflex on the left which was slightly diminished (1+). There was no decreased sensation to pin prick or vibration sense in either the upper or lower limbs; however, sensation to light touch was slightly decreased in the left L4 dermatome. Motor and cranial nerve exams were unremarkable. Blood pressure was 152/92 mmHg and the resting heart rate was 75 bpm (measured in the sitting position).

The left temporal headache and dizziness (uneasiness) could be reproduced by combined flexion and 25° of rotation of the head to the right. The same manoeuvre on the contralateral side was pain free at 50° of rotation. There was marked tenderness to palpation over the left C2/3 zygapophysial joint and the lumbar spinous processes.

On the basis of the clinical presentation, history, and findings of the physical examination, a working diagnosis of posterolateral L3/4 intervertebral disc herniation affecting the left L4 nerve root was made, and a trial of conservative management was suggested to the patient. The patient also received a concurrent working diagnosis of cervicogenic headache. In addition, the patient was recommended to seek further evaluation of his blood pressure.

### Case management and outcome

Conservative management consisted of cryotherapy (ice packs), high-velocity, low-amplitude spinal manipulation to the lumbar region, isometric stretching, and neurodynamic mobilisation techniques (neural flossing). This passive care was complemented by ergonomic advice and a prescribed back extension exercise program. The patient received a total of 6 treatments over a 4-week period. After this treatment regimen the patient reported the LBP to be 1-2/10 VAS, with no pain radiating down the left leg, and that he was able to perform all activities of daily living. Neurodynamic tension tests (e.g. straight leg raise and Bragard’s test) were negative. Lumbar spine ROM was improved (finger-to-floor reach 34 cm below the knees during flexion) and pain free.

The patient was discharged with a 4-week core muscle strengthening program and asked to return for a follow-up consultation after its completion, or earlier if symptoms returned. The patient returned asymptomatic after 4 weeks, and was able to satisfactorily perform the functional core strength tests.

### Second presentation

Two months after the last follow-up of the first episode of LBP, the patient presented to the chiropractic clinic with a new episode of acute LBP with pain radiating down the left leg. Similar to the first episode, the patient reported that the LBP had come on after an 8-h shift of driving a patrol car. However, this time the leg pain was radiating down the anterior aspect of the left thigh to the knee. He was taking prescription pain killers as directed by his general practitioner (1–2 tablets of 5 mg oxycodone [Endone, Aspen Pharamceuticals] per 6 h). However, the medication provided minimal relief and the pain was unrelenting, worse at night, and interfering with his sleep. Bladder and bowel function was normal. The physical examination mirrored the initial presentation but for two findings: (1) light touch was decreased in the left L3 and L4 dermatomes, and (2) slump test produced left anterior thigh pain radiating down to the left knee and lateral leg to the foot.

Because the patient now presented with indications of the possible involvement of multiple lumbar nerve roots, severe pain waking him up at night, and not responding to strong prescription pain medication, he was referred for magnetic resonance imaging (MRI) for an evaluation of possible compression of neurological structures at the L2 to L4 vertebral levels.

### Imaging

**Fig. 1:**
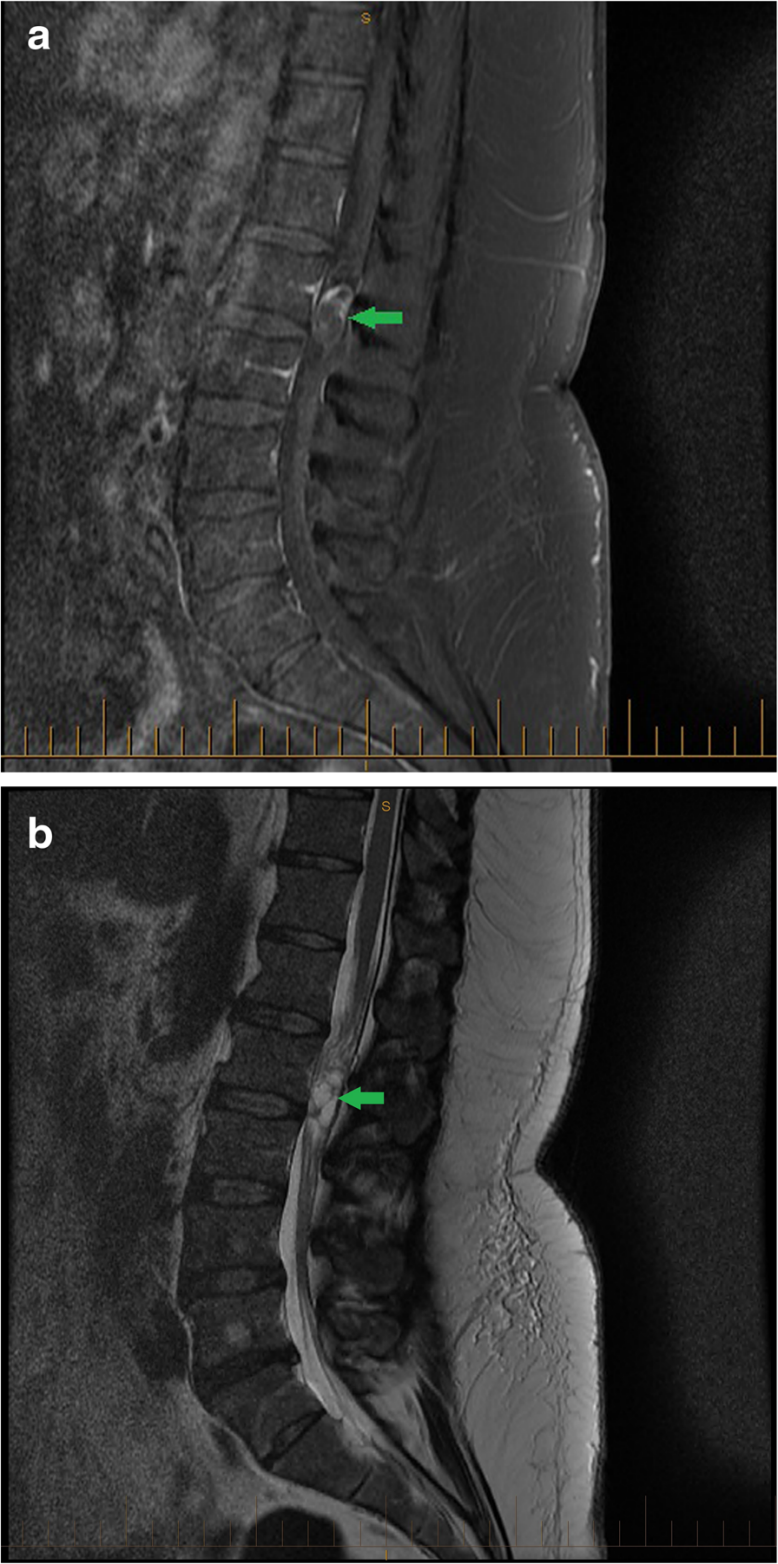
Sagittal fat suppressed T1-weighted (**a**) and T2-weighted (**b**) magnetic resonance images of the lumbosacral spine. A heterogeneous, predominantly cystic, intramedullary space-occupying lesion measuring 2.5 × 1.2 cm is present within the filum terminale at L2/3 level (green arrow). Additional findings include a transitional lumbosacral vertebra (lumbarisation of S1), dehydration of the L5/S1 intervertebral disc with a posterior disc bulge, and a hemangioma in the vertebral body of L5. The remainder of the spinal cord and conus medullaris, lower thoracic and lumbosacral vertebrae, intervertebral disc spaces, and paraspinal soft tissues appear normal

The MRI revealed a substantial heterogeneous space-occupying lesion with an intramedullary orientation within the filum terminale at the L2/3 vertebral level (see Fig. 1). The lesion measured approximately 2.5 cm × 1.2 cm in the craniocaudal and anterior-posterior dimensions, respectively. It appeared predominantly cystic on T2-weighted sequences and contained several fine internal septations. On post-contrast imaging, there was a heterogeneous enhancement of the lesion (predominantly peripheral), with a non-enhancing central aspect indicating a possible haemorrhagic or calcific component. The remainder of the spinal cord and conus medullaris appeared normal. Because the observed lesion most likely represented a myxoapapillary ependymoma, the patient was promptly referred for a neurosurgical consultation.

### Surgical intervention and outcome

The patient was scheduled for surgery the day after the neurosurgical consultation. A gross total resection was achieved and radiological adjunctive therapy was not pursued. Although the patient reported resolution of the neurological symptoms immediately after the surgery, low-grade LBP (3/10 VAS), described as a dull ache, still persisted for several weeks post-surgery. The patient received standard, post-surgical rehabilitation with a hospital-based physiotherapist for 6 months post-surgery. In long-term follow-ups at 6 and 12 months post-surgery, the patient reported no pain (0/10 VAS), with only a vague sensation of persistent numbness in the left anterior leg.

## Discussion

The present case highlights a number of important points: (1) the primary symptoms of intramedullary ependymomas of the caudal spinal cord can mimic ordinary LBP presentations, in particular lumbar intervertebral disc herniations; (2) clinicians need to be sensitive to subtle changes in the clinical presentation of LBP patients; and (3) prompt referral to advanced medical imaging such as MRI and early surgical intervention is key to achieve best possible outcomes for patients like the one described in the present case.

LBP is a very common health problem, and one of the leading causes of activity limitation and work absence worldwide [6, 7]. It is not surprising, therefore, that LBP is the most common complaint presenting to chiropractic clinics [8]. Although specific structural pathologies (e.g., intervertebral disk herniation or spinal stenosis) can be identified in a subset of patients presenting with LBP, the majority of cases (approximately 85 %) are referred to as nonspecific LBP [6, 9]. In the present case, the patient presented with typical LBP symptomatology with clinical findings indicative of a possible posterolateral L3/4 intervertebral disk herniation affecting the left L4 nerve root. Current evidence-based clinical practice guidelines recommend against the routine use of imaging in patients presenting with LBP [10, 11]. The patient was therefore offered conservative treatment consisting of a combination of passive and active care, to which he responded very well and was subsequently discharged.

At first glance the second episode appeared to be a typical recurrence of the first episode of LBP, with both the precipitating event and the clinical presentation of the second episode being very similar to the former. Furthermore, successful conservative management of the first episode could potentially lead to confirmation bias, that is, clinicians are likely to believe their working diagnoses are correct when their patients respond satisfactorily to care [12]. In the present case, however, a careful physical examination revealed subtle changes indicating the possible involvement of multiple lumbar spinal nerve roots. Moreover, the patient was not responding to strong prescription pain medication and the LBP would occasionally wake him up at night. Taken together, these were compelling reasons to suspect the presence of space-occupying pathologies such as spinal cord tumours and the patient was therefore referred to medical imaging.

It is difficult to estimate how often patients with an undiagnosed spinal ependymoma present to chiropractors with LBP because only a couple of cases have been reported in the literature to date. Lensgraf and Young described two cases: (1) a 46-year-old woman with 3–4 year history of intermittent LBP who eventually did not respond to conservative care; and (2) a 38-year-old man who presented with an unusual episode of LBP with leg pain [13]. In both cases the patient underwent surgical resection and was reported to be doing well at 12 months post-surgery [13]. Only the female patient received adjuvant radiation therapy [13].

In most jurisdictions, chiropractors can request advanced diagnostic imaging such as MRI. However, full government rebates for such referrals are only available in a few jurisdictions (e.g. Norway and Denmark). In Australia (the location of the present case), patients referred to MRI by chiropractors are not eligible for a Medicare rebate. Although the patient in the present case was both willing and able to pay for the MRI, this may of course not be true for all, or even the majority of, patients. It is, therefore, conceivable that such restrictions on advanced medical imaging referrals can contribute to delayed diagnosis of some patients.

Early detection and treatment is key to achieve best possible patient outcomes for people with spinal intramedullary ependymoma. These neoplasms are best treated surgically and a complete resection indicates complete resolution for the vast majority of patients [14, 15]. Adjuvant radiation therapy is usually reserved for cases where only subtotal resection is possible, while chemotherapy is used more sparingly, for instance in young children who are more prone to negative side effects from radiation therapy [16]. Surgery should be performed early because patients with a shorter duration of symptoms and less preoperative neurologic deficit tend to have better postoperative outcomes [2, 3, 13, 15]. Although the mean duration of symptoms in patients with spinal ependymoma is more than 3 years [2, 5], the patient in the present case was diagnosed and referred for neurosurgical consultation within 6 months of onset of initial symptoms.

## Conclusions

The primary symptoms of spinal intramedullary ependymomas can mimic ordinary LBP, in particular lumbar intervertebral disc herniations. Clinicians need to be sensitive to subtle changes in the clinical presentation of LBP patients. Prompt referral to advanced medical imaging such as MRI and early surgical intervention is key to achieve best possible outcomes for patients with spinal intramedullary ependymomas.

### Ethics and consent

This study was approved by the Human Research Ethics Committee at Central Queensland University (ethics approval reference number: H15/08–194). Written informed consent was obtained from the patient for publication of this case report and any accompanying images. A copy of the written consent is available for review by the Editor-in-Chief of this journal.
